# Comparison of EEG measurement of upper limb movement in motor imagery training system

**DOI:** 10.1186/s12938-018-0534-0

**Published:** 2018-08-02

**Authors:** Arpa Suwannarat, Setha Pan-ngum, Pasin Israsena

**Affiliations:** 1 Department of Computer Engineering, Faculty of Engineering, Chulalongkorn University, Phayathai Road, Wang Mai, Pathumwan, Bangkok, 10330 Thailand; 2 National Electronics and Computer Technology Center, 112 Thailand Science Park, Phahonyothin Road, Khlong Nueng, Khlong Luang, Pathumthani, 12120 Thailand

**Keywords:** Rehabilitation technology, Brain computer interface (BCI), Common spatial pattern (CSP), Motor imagery (MI)

## Abstract

**Background:**

One of the most promising applications for electroencephalogram (EEG)-based brain computer interface is for stroke rehabilitation. Implemented as a standalone motor imagery (MI) training system or as part of a rehabilitation robotic system, many studies have shown benefits of using them to restore motor control in stroke patients. Hand movements have widely been chosen as MI tasks. Although potentially more challenging to analyze, wrist and forearm movement such as wrist flexion/extension and forearm pronation/supination should also be considered for MI tasks, because these movements are part of the main exercises given to patients in conventional stroke rehabilitation. This paper will evaluate the effectiveness of such movements for MI tasks.

**Methods:**

Three hand and wrist movement tasks which were hand opening/closing, wrist flexion/extension and forearm pronation/supination were chosen as motor imagery tasks for both hands. Eleven subjects participated in the experiment. All of them completed hand opening/closing task session. Ten subjects completed two MI task sessions which were hand opening/closing and wrist flexion/extension. Five subjects completed all three MI tasks sessions. Each MI task comprised 8 sessions spanning a 4 weeks period. For classification, feature extraction based on common spatial pattern (CSP) algorithm was used. Two types were implemented, one with conventional CSP (termed WB) and one with an increase number of features achieved by filtering EEG data into five bands (termed FB). Classification was done by linear discriminant analysis (LDA) and support vector machine (SVM).

**Results:**

Eight-fold cross validation was applied on EEG data. LDA and SVM gave comparable classification accuracy. FB achieved significantly higher classification accuracy compared to WB. The accuracy of classifying wrist flexion/extension task were higher than that of classifying hand opening/closing task in all subjects. Classifying forearm pronation/supination task achieved higher accuracy than classifying hand opening/closing task in most subjects but achieved lower accuracy than classifying wrist flexion/extension task in all subjects. Significant improvements of classification accuracy were found in nine subjects when considering individual sessions of experiments of all MI tasks. The results of classifying hand opening/closing task and wrist flexion/extension task were comparable to the results of classifying hand opening/closing task and forearm pronation/supination task. Classification accuracy of wrist flexion/extension task and forearm pronation/supination task was lower than those of hand movement tasks and wrist movement tasks.

**Conclusion:**

High classification accuracy of the three MI tasks support the possibility of using EEG-based stroke rehabilitation system with these movements. Either LDA or SVM can equally be chosen as a classifier since the difference of their accuracies is not statistically significant. Significantly higher classification accuracy made FB more suitable for classifying MI task compared to WB. More training sessions could potentially lead to better accuracy as evident in most subjects in this experiment.

## Background

Brain computer interface (BCI) is an emerging technology that provides alternative ways of communication between human and environment or devices. Applications range from real physical device control to user interactive such as game play. One of non-invasive BCI technologies is electroencephalography (EEG). EEG has been widely used due to its desirable properties. It is relatively low cost and also relatively easy to install [1, 2].

The brain rhythms which have been used in EEG-based BCI studies are sensorimotor rhythms (SMRs) which occur on the motor cortex area of the brain [1, 2]. Alpha/mu band (8–13 Hz) and beta band (13–30 Hz) are the frequency bands of SMRs. Movements or imagination of motor action which is called motor imagery (MI) lead to the changes in SMRs. The phenomena called event-related de-synchronization (ERD) and event-related synchronization (ERS) are the result of the change [3].

Stroke is one of severe neurological impairments that BCI technology has been applied to [4–8]. Among world populations, stroke leads to cause of death and various disabilities such as the lack of fully functional arm, wrist or hand. The loss of quality of life is the result of these disabilities [9–12]. Therefore, EEG-based stroke rehabilitation is one of the most interesting applications for BCI technology. To regain some functional controls in stroke patients is the purpose of the application. The potential of BCI technology that might help to restore motor control in stroke patients is supported by many studies [10–15]. Robot-assisted EEG-based rehabilitation has also received a lot of attention [16–21]. The studies by Ang et al. showed that EEG-based technology achieved better results compared to traditional rehabilitation [16, 17]. Furthermore, robot-assisted EEG-based rehabilitation was found to achieve better results than EEG-based rehabilitation [16, 17]. These results were seen in the significant improvement of Fugl-Meyer motor assessment (FMMA) score which measures the capability of motor control [22].

The capability of detecting MI is one of the key points in development of EEG-based stroke rehabilitation application [23]. To achieve the goal, many feature extraction algorithms have been proposed. Among those algorithms, common spatial pattern (CSP) is the state-of-the-art algorithm [24, 25]. Hence, many algorithms that derived from CSP have also been proposed [26–31]. Filter bank common spatial pattern (FBCSP) is one of the algorithms that are derived from CSP [26, 27]. It is also one of the most popular feature extraction algorithms in detecting MI studies. The studies by Ang et al. showed that FBCSP achieve significantly higher accuracy compared to conventional CSP [26, 27]. Hence, applying the feature extraction algorithm is highly interesting due to it affects to MI detection accuracy. Most of MI-based BCI studies used hand opening/closing as MI task [16–18, 23–27]. Functional hand control is also the most widely used functional control in EEG-based stroke rehabilitation. The use of wrist movement task could be seen in few studies [28–32]. However, the use of wrist movements as MI task can be found in specific EEG-based stroke rehabilitation studies [17, 33, 34]. These studies demonstrated the feasibility to classify wrist flexion/extension, pronation/supination. The studies by Edelman et al. also showed the most discriminable features for each of the four MI tasks [31, 32]. This indicates that wrist and arm rehabilitations are also relevant.

Moreover, in EEG-based applications that control real or even virtual hardware, more MI tasks would provide more commands to control those output devices [35–37]. Typically, foot and tongue MI are first considered to increase the number of commands. However, It is not intuitive to control robotic arm using foot or tongue MI. Imagining the action of arm or hand to control robotic arm is more natural [32]. Accordingly, the EEG-based stroke rehabilitation application is not the only application that benefits from the study of using more complex MI. Other EEG-based applications such as device control would also benefit.

The use of EEG headset with minimal channels is also interesting in development of EEG-based stroke rehabilitation system. EEG headsets with high number of channels are used in many studies [16–21]. These researches show potential of BCI for stroke rehabilitation. From our experience, the setup of multi-channel headsets sometimes takes almost 1 h. Some headsets with wet electrodes could also make subjects irritate. Furthermore, from American electroencephalographic society guidelines in EEG [38], hand control could be detected from small area around the center of the scalp. It is thus interesting in explore EEG-based stroke rehabilitation system with minimal channels around this scalp area.

This study will evaluate the effectiveness of three movements of hand and wrist for MI tasks, which are the key exercises given to patients in conventional rehabilitation [39]. The objective of the study is to investigate the feasibility of experimental paradigm of upper limb MI training system. The paradigm would be then applied in development of an upper limb rehabilitation system with minimal channels for stroke patients. The system would finally be bundled with robotic arms that were published in [40, 41].

## Methods

### Motor imagery tasks

**Fig. 1:**
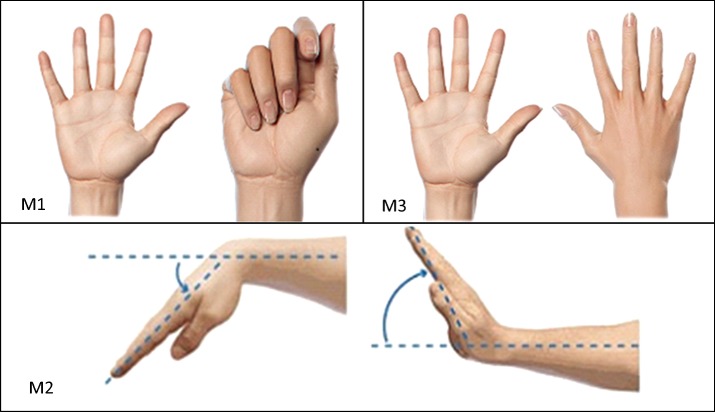
Three MI tasks. M1 is hand opening/closing task [62]. M2 is forearm pronation/supination task [62]. M3 is wrist flexion/extension task [63]

MI is the imagination of motor action [3]. Three MI tasks are chosen in this study. The tasks consist of hand opening/closing, wrist flexion/extension and forearm pronation/supination as shown in Fig. 1. These movements are mentioned in the clinical practice guideline for stroke rehabilitation [42]. They are also suggested in neurological rehabilitation [43]. Hand opening/closing and wrist flexion/extension are two of three key exercises given to patients for rehabilitation of the hand and wrist [44].

Hand opening/closing is a major MI task in MI-based BCI studies. Moreover, the movement is also one of the basic movements of stroke rehabilitation because it is the basic motion for grasping object [43, 44]. The subject was instructed to imagine of squeezing and releasing a tennis ball in his/her hand. Hand opening/closing is referred to as M1.

Wrist flexion/extension is the exercise that a patient should perform to regain full range of motion and use of wrist [44]. Wrist flexion is the movement of bending the palm down, towards the wrist. Wrist extension is the movement of raising the back of the hand, as shown in Fig. 1. A subject assumes a neutral or flat wrist position, then tilts his/her hand downwards as far as possible, with the maximum of 90° downwards in flexion motion. Extension motion also starts with flat wrist position, then the subject tilts his/her hand upwards as far as possible, with the maximum of raising the back of the hand 90° [44]. Wrist flexion/extension is referred to as M2.

Forearm pronation/supination is the movement that patients may be advised to carry out for rehabilitation although it is not one of the key exercises [44]. Forearm pronation is the movement of rotating the forearm into a palm down position. Forearm supination is the movement of rotating the forearm into a palm up position [44]. Thus, this task is the forearm rotation motion for approximately 180°. Forearm pronation/supination is referred to as M3.

### Subjects

Eleven healthy subjects participated in the study. All of the subjects were new to BCI usage. All subjects completed the experiment of hand opening/closing. Ten subjects completed the experiment of wrist flexion/extension. Five subjects completed the experiment of forearm pronation/supination.

### EEG data acquisition

EEG data was acquired using G.Nautilus headset [45] providing 16 Ag/AgCl electrodes positioned according to the 10/20 system [38]. The data was digitally sampled at 250 Hz.

According to the study by Yuan et al. [1], hand, wrist and arm movement cover the position of C3 and C4 to the center of scalp. Accordingly, apart from the positions of C3, Cz and C4 that are on the area, the adjacent positions which are F3, Fz, F4, P3, Pz, P4, T7 and T8 are also chosen. The EEG data was recorded from these eleven electrodes.

### EEG data analysis

**Fig. 2:**
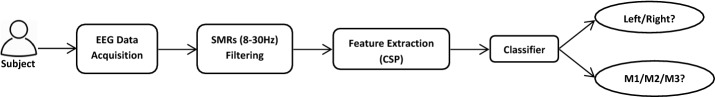
EEG Data analysis process

EEG data analysis process is illustrated in Fig. 2. The process is offline. It gives two types of classification. The first type is left hand and right hand classification of the same task. Classifying each MI task is the second type. According to the figure, M1 is hand opening/closing task. Wrist flexion/extension is referred to as M2 and forearm pronation/supination is referred to as M3.

**Fig. 3:**
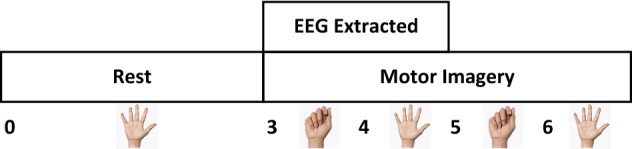
Experimental trial timeline

Recorded EEG data was processed in Matlab (The Mathworks Inc., Natick MA, USA). EEG data was extracted from the third and the fourth second from each trial according to Fig. 3 so that the extracted EEG data come from two motions of each task. The data was filtered from 8 to 30 Hz which is the SMRs rhythms. Feature extraction algorithm was then applied to the filtered data. Classifier finally processed the extracted features to give the classification results.

Paired t-test was performed to analyze the significant difference from baseline in the MI classification using LDA vs SVM, and WB feature vs FB feature (see Table 1). Analysis of variance (ANOVA) with Bonferroni correction was used to examine baseline differences between classification accuracy of the first session and the last session of each MI task. All data analysis was performed using SPSS (IBM Corp., New York, USA) and the level of significance was set at 5%.

**Table 1:** The results of comparing of classification accuracies of the use of LDA and SVM and the use of WB feature and FB feature

MI task	Classification accuracy (%) ± SD			
	LDA	SVM	WB	FB
Opening/closing	64.07 ± 7.81	64.02 ± 7.93	61.90 ± 6.32**	66.19 ± 8.64**
Flexion/extension	68.79 ± 10.71	68.71 ± 10.84	66.40 ± 9.70**	71.10 ± 11.26**
Pronation/supination	69.10 ± 10.62	68.97 ± 10.78	66.38 ± 9.59**	71.69 ± 11.08**

** P < 0.001

### Feature extraction

CSP which is the state-of-the-art feature extraction algorithm was used. CSP is based on statistical classification. Multichannel data is classified into two classes. The method of CSP could be briefly described in two steps in supervised manner [24]. In the first step, training data from two classes are processed. The result of the first step is spatial filter. To classify data, the filter transforms input data into feature space which discriminable features are the variances of the two classes of data. The variance of one class is maximized while the variance of another class is minimized [24]. The second step uses spatial filter to classify unknown class of new data.

In this study, two different types of brain rhythms or frequency band were extracted. The first type was applying CSP to extract feature from whole band of SMRs. In the second type, SMRs was filtered into five bands of brain rhythms which were 8–12, 12–16, 16–20, 20–24 and 24–30 Hz. CSP was then applied to those filtered SMRs. The first type was referred to as “Whole band” or “WB” and the second type was referred to as “Filter Bank” or “FB”.

In addition, unlike FBCSP as mentioned in [26, 27], the aim of FB in this study is just to study the effect of the increasing number of features to classification accuracy. FBCSP has a feature selection algorithm which is Naïve Bayes Parzen Window (NBPW) while FB has no such an algorithm.

### Classifiers

In MI-based BCI studies, linear classifiers are more widely used than non-linear classifiers [46]. EEG signals are noisy and non-stationary which are high dimensionality and high variance [47]. Number of parameters of linear classifiers is less than that of non-linear classifiers. Although this may lead to overfitting, the problem could be handled with regularization. Due to fewer number of parameters, linear classifiers take less computational time and memory [48]. Linear discriminant analysis (LDA) is one of linear classifiers. It is the most popular linear classifier in MI-based BCI research [39, 46].

Support vector machines (SVM) has desirable properties to deal with EEG signals. These properties are noise tolerance and high-dimensionality robustness. Thus, SVM is suitable for EEG which is noisy, non-stationary and high variance signal [47].

Consequently, LDA and SVM are the two classifiers that are chosen in this study.

### Experimental paradigm

During the trials, subjects sat comfortably facing a computer screen and were instructed to perform MI tasks of right hand and left hand respectively. The experimental session of each MI task consisted of eight sessions. Each session comprised eight runs of EEG data collection. Subjects performed right hand MI for the first four runs and performed left hand MI for the last four runs. Each runs comprised twenty trials. Each trial lasted 7 s as outlined in Fig. 3.

**Fig. 4:**
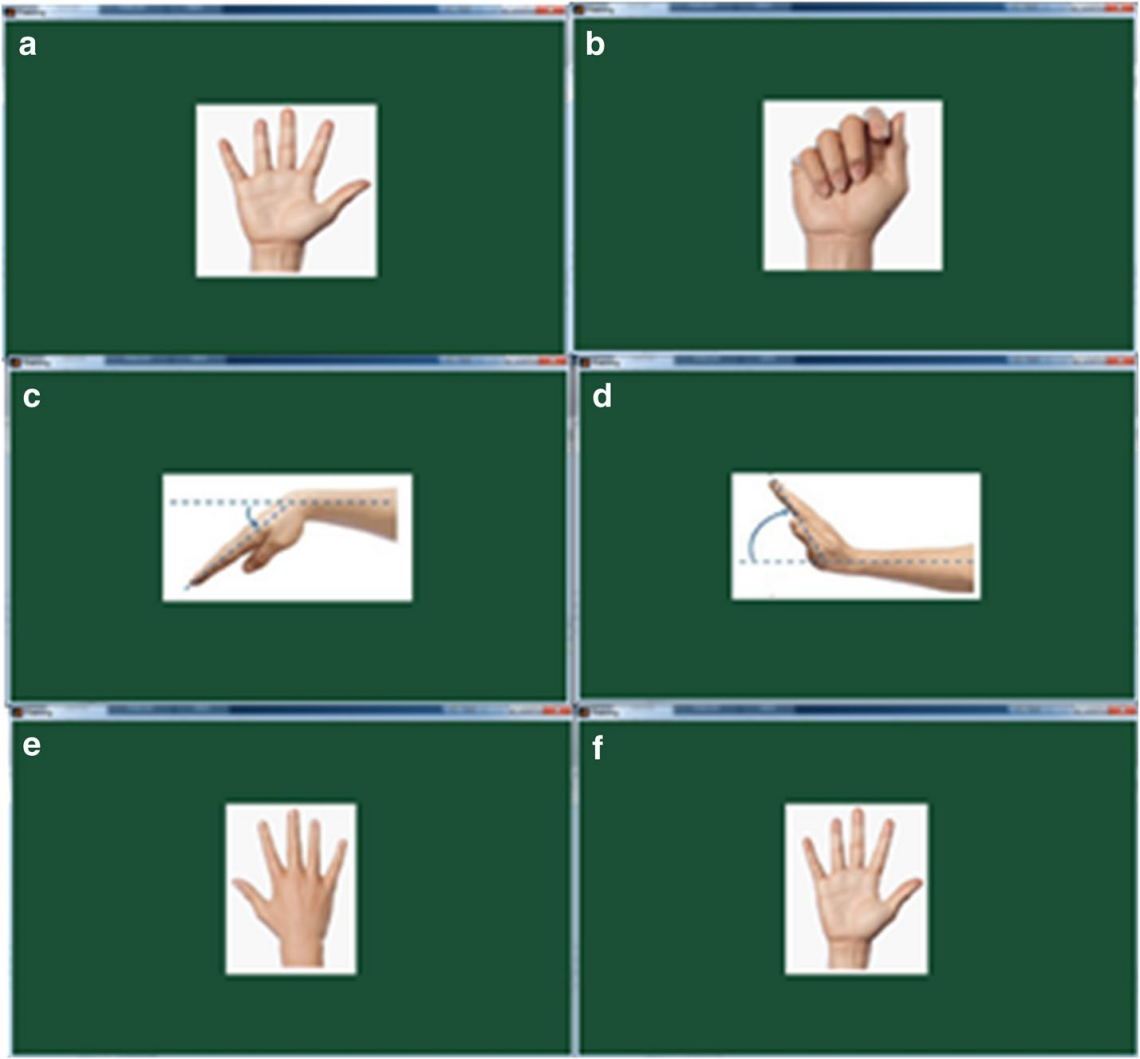
Display screen. **a**, **b** screen of hand opening/closing task. **c**, **d** screen of forearm pronation/supination task. **e**, **f** screen wrist flexion/extension task

Instructions to subjects and notification screen are designed based on BCI2000 which is a software suite for EEG research [49]. BCI2000 was used to record and process EEG data in many studies [35–37, 50]. The experimental paradigm in BCI2000 for Mu rhythms is called Stimulus Presentation. The experiment uses blank screen for rest state and uses left arrow or right arrow for left MI or right MI. A subject is instructed to relax or stop movement imagery when blank screen is displayed. When left arrow or right arrow is displayed, subject is instructed to imagine movement of respective hand [51]. Accordingly, blank screen is displayed in rest period and left arrow or right arrow is displayed in motor imagery period. In our experimental paradigm blank screen and left arrow or right arrow are replaced by the picture of hand movements in Fig. 4.

According to Fig. 3, in hand opening/closing task, subject was instructed to perform hand opening in the first 3 s. Hand opening and hand closing were then alternately performed in last 4 s. Pictures of hand movements as shown in Fig. 4 was used to notify the subject. During the task, the subject was only notified twice. The picture of hand opening was on display during the first 3 s. To avoid the effect of visual observation on EEG, in the last 4 s, only the picture of hand closing was constantly displayed. During those 4 s subject did the hand opening/closing tasks alternately every second by his/her own estimate.

The other two MI tasks experiments were conducted in the same manner. Consequently, each session took approximately 1 h including set-up time.

Each subject participated in the experiment 2 sessions/week. The experiment of each MI was completed in 4 weeks. Hand opening/closing was the first task for the experimental session. The second task was wrist flexion/extension. Forearm pronation/supination was the last task of the experiment. With three MI, the experiment was completed in 12 weeks.

## Results

For each session of the three tasks, a subject had to sit through a 10–30 min EEG measurement setup to achieve good quality signals. The experiment session lasted 40 min, so overall each session took approximately 1 h. Each subject did 2 sessions/week for 4 consecutive weeks for one task. Hence subjects who did all three tasks spent 3 months doing the experiments. Because of this long duration and the setup process, some subjects dropped out of the subsequent tasks.

Results are shown in classification accuracies of classifying left and right hand of each MI task. Classification accuracy reflects the subject’s ability to perform an MI task. The accuracies are the results from a set of classification parameters which were session dependent training and using EEGs from all eleven electrodes. They were calculated using eightfold cross validation method.

The classification in this study was binary classification. Two types of MI classification were mentioned. The first classification was the classification of left hand MI vs right hand MI of each task. The second classification was the classification of two MI tasks of left hand or right hand. For instance, the classification of hand opening/closing of left hand MI vs wrist flexion/extension of left hand MI.

The results of the three MI tasks were calculated and analyzed to compare the followings: performance of the two classifiers, LDA and SVM; performance of the two feature extraction methods, WB and FB; performance trends of subjects through training sessions; performance of subjects on the three MI tasks.

### Comparison of classifiers

The use of LDA and SVM as a classifier is the first comparison. The classification accuracies of classifying left hand MI vs right hand MI of each task were assessed in the comparison as shown in Table 1.

**Fig. 5:**
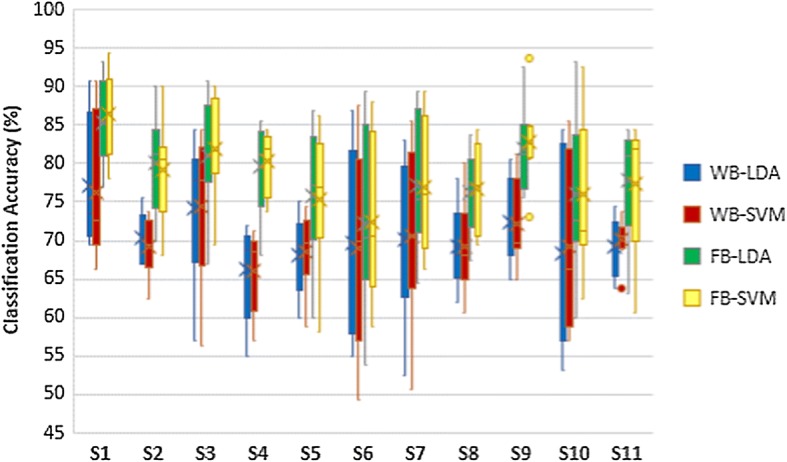
Classification accuracies of hand opening/closing task

**Fig. 6:**
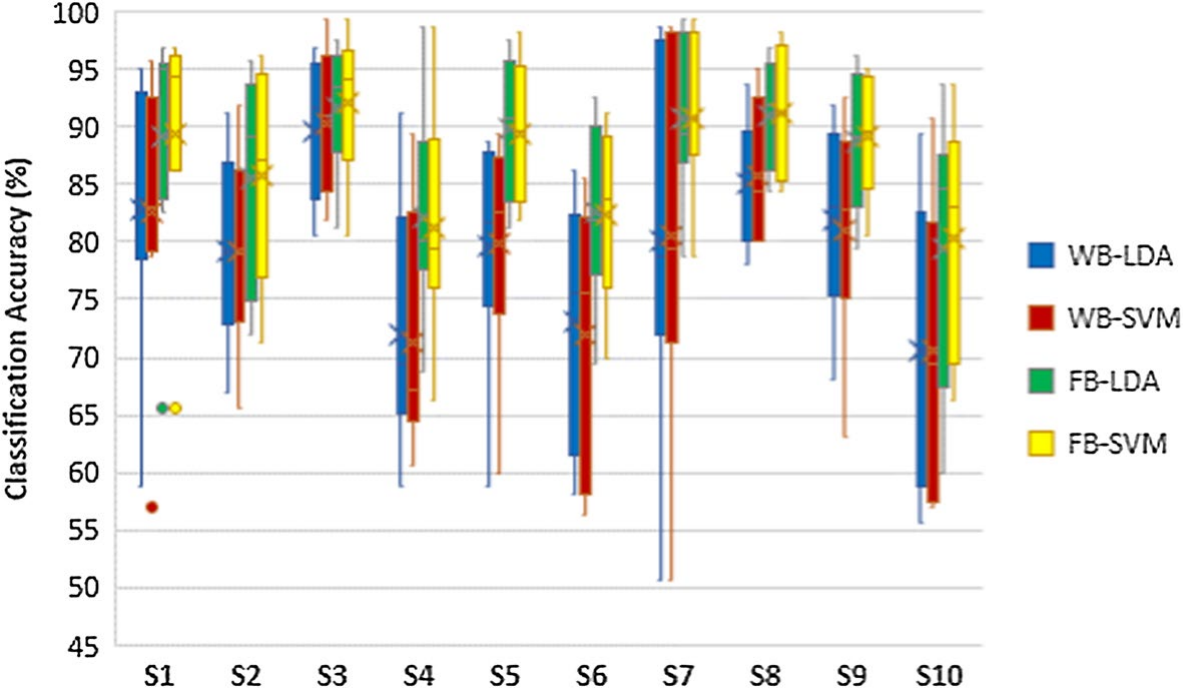
Classification accuracies of wrist flexion/extension task

**Fig. 7:**
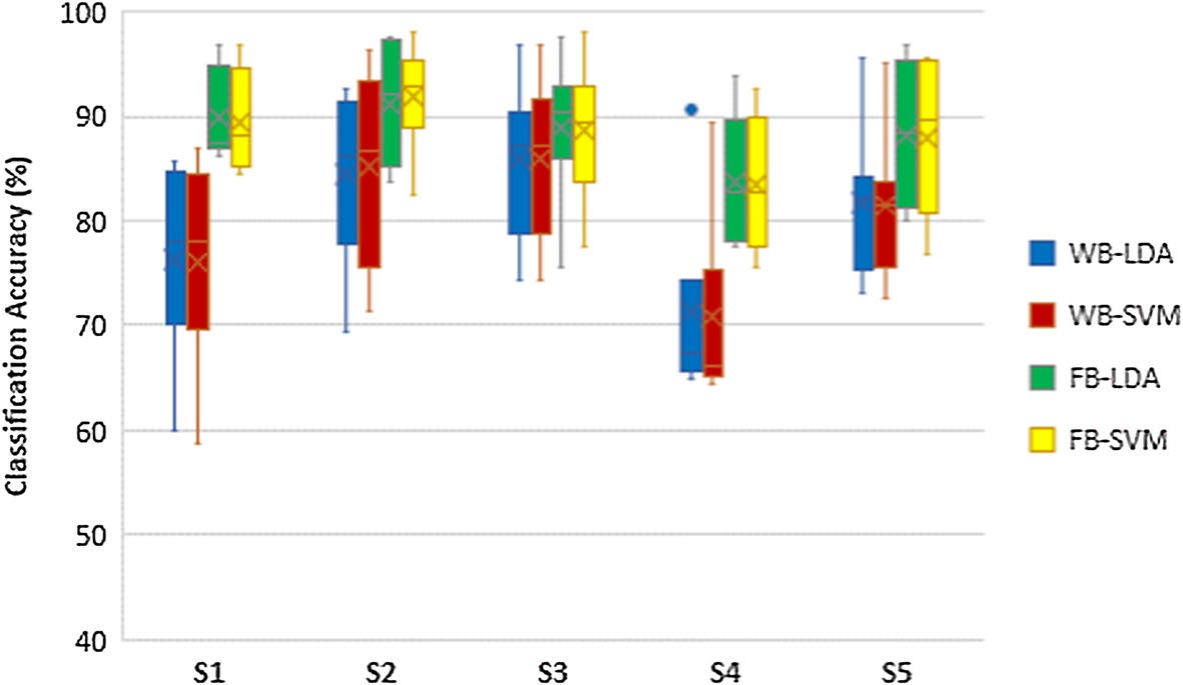
Classification accuracies of forearm pronation/supination task

The results of classifier comparison of hand opening/closing task are shown in Fig. 5. Wrist flexion/extension results and forearm pronation/supination results are shown in Figs. 6 and 7 respectively. Blue plots represented using LDA with WB feature. Green plots represented using LDA with FB feature. Using SVM with WB feature and FB feature were represented by red plots and yellow plots respectively. According to those figures, the pairs of blue-red plots and green-yellow plots are comparable. Thus, there are no statistically significant differences in classification accuracy between LDA and SVM in each task.

### Comparison of feature extraction methods

The comparisons of classification accuracy of using WB and FB as a feature extraction are also shown in Figs. 5, 6 and 7. The classification accuracy of classifying left hand MI vs right hand MI of each task were assessed in the comparison as shown in Table 1. The yellow plot is much higher than red plot, while the green plot is much higher than the blue plot. The results demonstrate that FB feature achieves higher classification accuracy compared to WB features in all tasks of MI. The column 3 and 4 of Table 1 show that the higher accuracies of FB is statistically significant.

### Trend analysis

The classification accuracy could be considered the capability of performing MI of subject. As mentioned in the studies by Ang et al. [16, 17], the capability of performing MI could be increased with more sessions of experiments. Thus, classification accuracies of individual sessions were observed and analyzed.

**Fig. 8:**
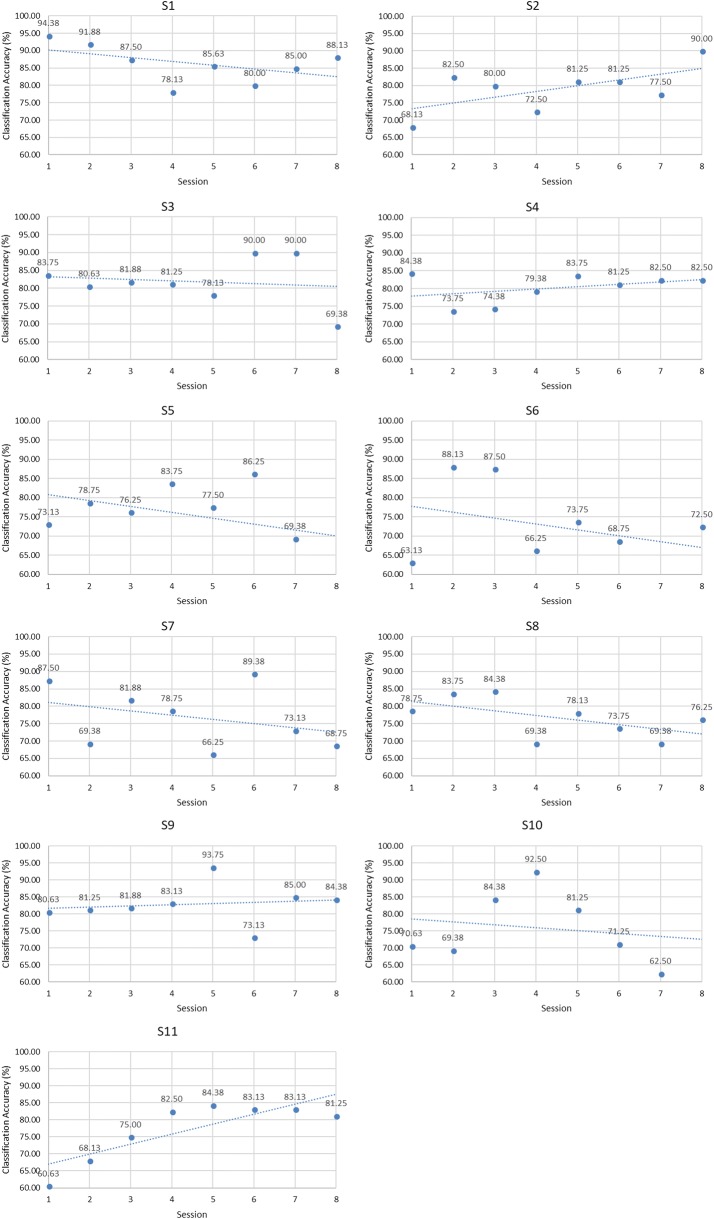
Trend analysis of hand opening/closing task

**Fig. 9:**
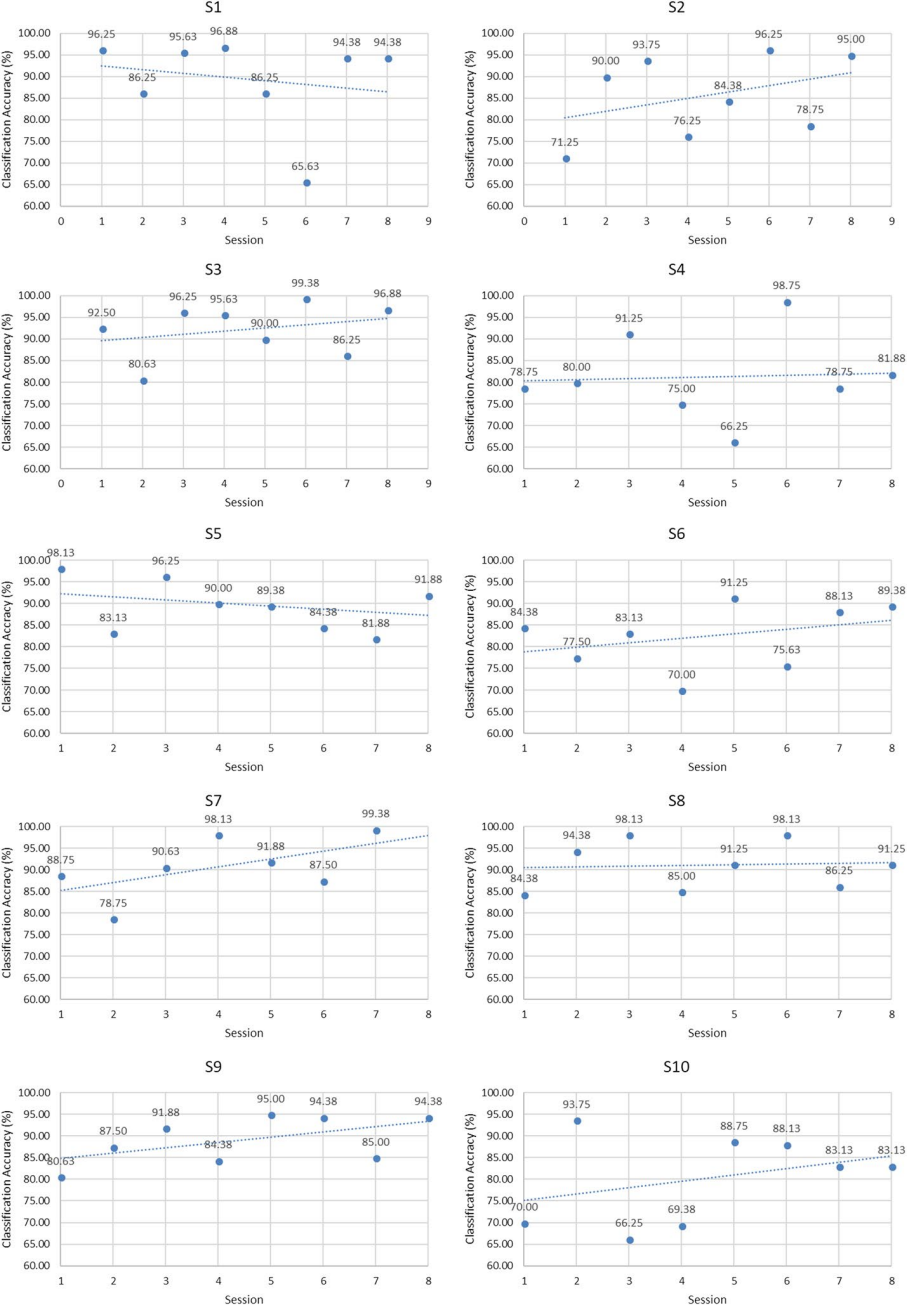
Trend analysis of wrist flexion/extension task

**Fig. 10:**
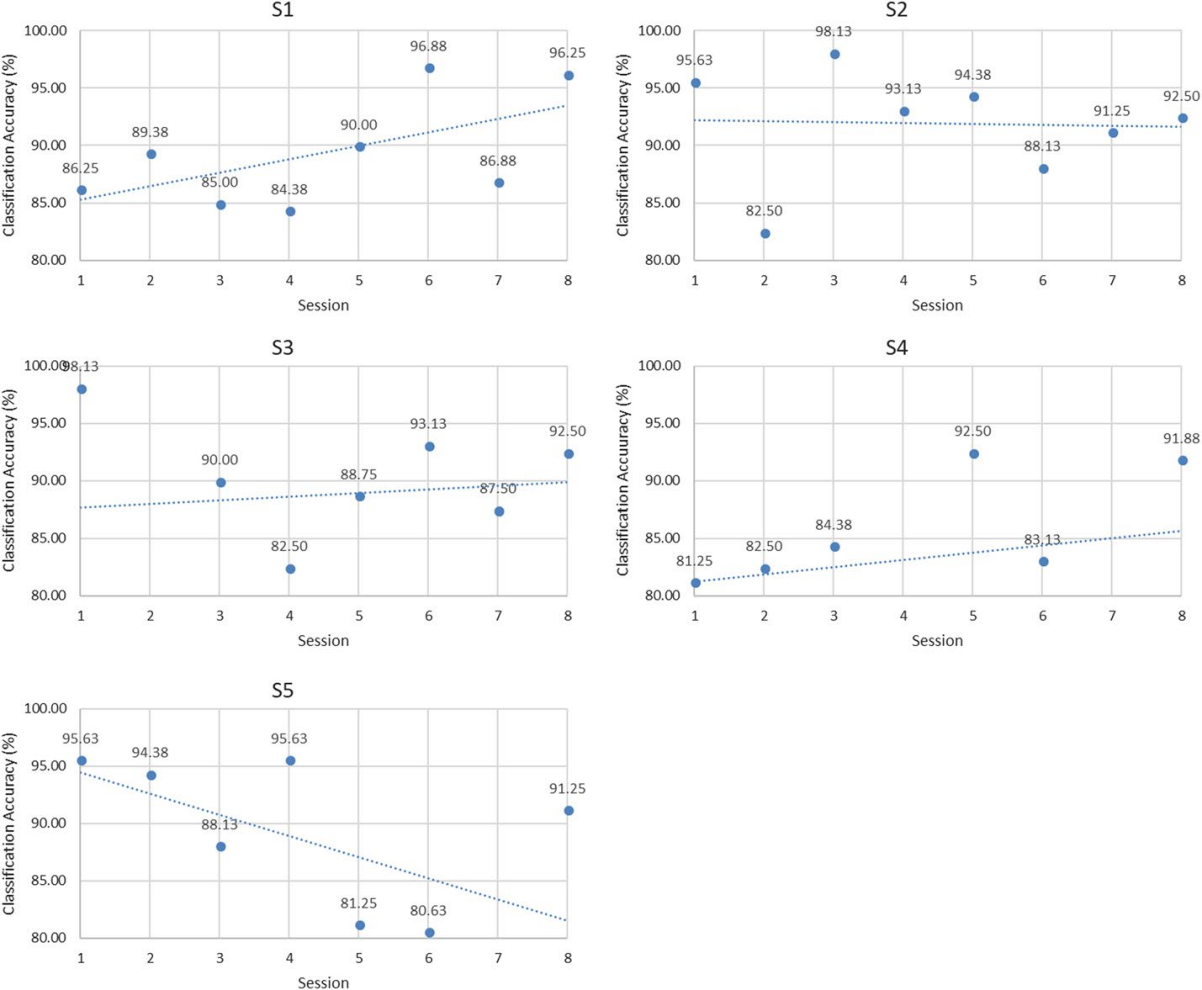
Trend analysis of forearm pronation/supination task

According to the results of classifier and feature, the accuracies of individual sessions of each MI task were demonstrated in Figs. 8, 9 and 10 when the classifier was SVM and FB feature was used.

Trend analysis of classification accuracy of individual sessions was performed using Microsoft Excel as shown in Table 2. Trends of hand opening/closing task are shown in Fig. 8. Trends of wrist flexion/extension are shown in Fig. 9 whereas Fig. 10 demonstrated the trends of forearm pronation/supination task.

**Table 2:** Classification accuracies of the first session and the differences of the classification accuracies from the last session of each MI task

Subject	Differences of classification accuracies (%)		
	M1	M2	M
S1	− 6.56	− 1.25	+ 10.00**
S2	+ 20.94**	+ 22.50**	− 4.69
S3	− 15.62	+ 4.06	− 5.31
S4	− 3.44	+ 2.81	+ 10.31**
S5	− 13.75	− 4.69	− 4.69
S6	+ 8.75*	+ 5.63*	–
S7	− 18.75	+ 10.63**	–
S8	+ 1.56	+ 7.19*	–
S9	+ 6.87	+ 14.06**	–
S10	− 10.31	+ 12.81*	–
S11	+ 18.75**	–	–

* P < 0.05, ** P < 0.001

In hand opening/closing task, an upward trend could be seen in four subjects (S2, S4, S9 and S11) while the opposite trend could be seen in seven subjects (S1, S3, S5, S6, S7, S8 and S10).

In wrist flexion/extension task, an upward trend could be seen in eight subjects (S2, S3, S4, S6, S7, S8, S9 and S10). The downward trend could be seen in two subjects (S1 and S5).

In forearm pronation/supination task, the upward trend could be seen in three subjects (S1, S3 and S4) while the downward trend could be seen in two subjects (S2 and S5).

Moreover, although downward trend was seen in some subjects, upward trend could also be seen in some periods of experimental session.

In conclusion, from all experiments of MI tasks, the upward trend of classification accuracy of individual sessions was found in 10 subjects (S1, S2, S3, S4, S6, S7, S8, S9, S10 and S11). S5 was the only subject that did not achieve upward trend in all MI tasks.

### Classifying each MI task

Accuracy of classifying each MI task of left and right hand are also observed. For instance, the classification of hand opening/closing of left hand MI vs wrist flexion/extension of left hand MI. The accuracy was calculated from data from all sessions using eight-fold cross validation method. The results are shown in Table 3. Three pairs of movements were grouped because CSP is the feature extraction algorithm that is suitable for classifying between two classes. Moreover, LDA and SVM are binary classifiers.

**Table 3:** Accuracies of classifying each MI task

Subject	Feature	Hand	M1M2		M1M3		M2M3	
			LDA	SVM	LDA	SVM	LDA	SVM
S1	WB	Left	83.67	83.67	85.00	85.39	65.16	68.20
		Right	83.05	83.36	83.75	83.59	73.83	74.45
	FB	Left	87.58	87.11	88.28	88.44	73.13	73.05
		Right	85.31	85.39	83.75	83.05	79.53	79.77
S2	WB	Left	78.67	78.44	77.89	78.13	68.28	67.58
		Right	79.30	79.30	80.39	79.30	74.06	75.39
	FB	Left	83.67	83.75	84.30	84.38	78.75	79.61
		Right	85.63	86.33	86.02	86.25	81.41	81.41
S3	WB	Left	78.67	78.44	77.89	78.13	68.28	67.58
		Right	79.30	79.30	80.39	79.30	74.06	75.39
	FB	Left	83.67	83.75	84.30	84.38	78.75	79.61
		Right	85.63	86.33	86.02	86.25	81.41	81.41
S4	WB	Left	94.06	94.38	92.89	93.59	69.06	69.06
		Right	87.73	89.45	93.28	93.91	69.30	69.38
	FB	Left	94.61	94.84	96.09	96.25	74.30	73.44
		Right	94.22	94.61	96.80	96.80	78.05	78.44
S5	WB	Left	75.47	75.70	83.98	84.69	72.81	71.17
		Right	74.22	74.77	77.58	77.50	80.08	79.14
	FB	Left	78.05	78.13	89.14	88.28	81.95	83.36
		Right	80.08	80.55	85.31	85.63	84.14	84.30
S6	WB	Left	82.42	82.11	–	–	–	–
		Right	74.22	73.36	–	–	–	–
	FB	Left	87.34	88.36	–	–	–	–
		Right	74.22	73.75	–	–	–	–
S7	WB	Left	82.41	82.95	–	–	–	–
		Right	82.32	82.86	–	–	–	–
	FB	Left	85.27	85.36	–	–	–	–
		Right	87.59	89.46	–	–	–	–
S8	WB	Left	77.34	78.75	–	–	–	–
		Right	80.86	80.78	–	–	–	–
	FB	Left	83.36	85.23	–	–	–	–
		Right	87.03	86.95	–	–	–	–
S9	WB	Left	72.32	72.05	–	–	–	–
		Right	78.66	78.48	–	–	–	–
	FB	Left	85.45	85.45	–	–	–	–
		Right	83.75	84.29	–	–	–	–
S10	WB	Left	62.77	62.68	–	–	–	–
		Right	71.70	71.96	–	–	–	–
	FB	Left	76.34	76.88	–	–	–	–
		Right	80.71	80.98	–	–	–	–

According to Table 3, M1 represents hand opening/closing task. M2 represents wrist flexion/extension task while forearm pronation/supination was referred to as M3. Thus, M1M2 meant the classification of hand opening/closing task and wrist flexion/extension task. M1M3 indicated the classification of hand opening/closing and forearm pronation/supination. The classification of wrist flexion/extension and forearm pronation/supination was referred as M2M3. The results are the mean classification accuracy of all sessions of classifying each MI task of left hand and right hand.

In M1M2, one subject achieved higher than 90% accuracy. Three subjects achieved lower than 80% in one hand whereas the accuracies were between 80 and 90% in the rest of subjects. The accuracies were comparable when using LDA and SVM. FB feature achieve higher accuracy than WB feature. There were no differences between accuracy of left and right hand in most subjects.

The results of M1M3 and M2M3 were similar to the results of M1M2. The use of LDA and SVM yielded comparable classification accuracy. FB feature achieved higher classification accuracy compared to WB feature. The accuracies of left and right hand were also comparable. Furthermore, the results of M1M2 and M1M3 were higher than the results of M2M3. The accuracies of M1M2 and M1M3 were between 80 and 90% in most subjects while the accuracies of M2M3 were approximately 70–80%.

## Discussion

Healthy subjects were recruited because ERD and ERS are the phenomena that occur to both healthy and stroke patients [3]. Healthy subjects usually show activation in motor imagery on the opposite side of the brain (contralateral activation) [3, 52]. Channel selection in a stroke rehabilitation study using BCI was performed by Buch et al. [50]. The results show that some subjects had their most effective channels for control on the opposite side (contralateral activation) while some had them on the same side (ipsilateral activation) [50]. The activation on the same side is also reported in stroke recovery in fMRI studies [53]. This is similar to the results in the study by Tam et al. which found that stroke patients had their own individual activation patterns [54]. Furthermore, in stroke patients, activation of the frontal premotor area and parietal area during motor imagery has also been reported [55]. The study of Ang et al. also stated that the neurological damage to the brain of stroke patients does not significantly affect their capability of operating MI-BCI [56]. ERD study by Stępień et al. also shows that there was no significant ERD difference between the subcortical stroke patients and control group (healthy subjects) [57]. Furthermore Gomez-Rodriguez et al. [20] carried out MI experiments on both stroke patients and healthy subjects and concluded that haptic feedback activates the somatosensory cortex in stroke patients as well as in healthy subjects.

Although, in the study by Kasashima et al. the finding means that ERD baseline in stroke patients is relatively lower than that in healthy subjects [58]. However, the problem could be handled by using feature extraction techniques such as CSP which is realized by projections of the high-dimensional, spatial–temporal raw signals onto very few specifically designed spatial filters. These filters are designed in such a way that the variances of the input signals carry the most discriminative information [24]. Hence, the EEG-based BCI system that uses CSP as feature extraction technique has potential to classify MI tasks accurately in both healthy subjects and stroke patients. Furthermore, Xu et al. [19] also reports development of stroke rehabilitation system on healthy subjects.

To compare the differences of classification accuracies of using LDA and SVM and the differences of classification accuracies of using WB feature and FB feature, Paired t-test was performed. P-value < 0.05 indicates statistically significant difference between tasks. Classification accuracies of classifying of left hand MI and right hand MI with standard deviation are demonstrated.

**Fig. 11:**
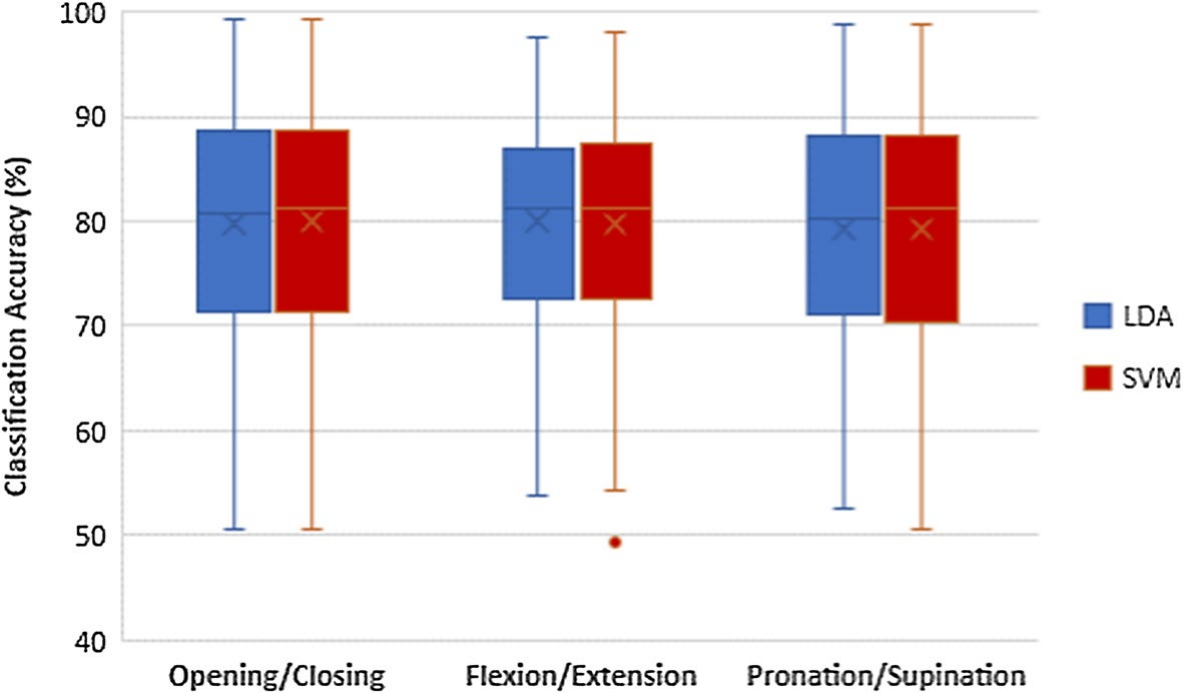
Comparison of LDA and SVM

According to Fig. 11, there are no statistically significant differences in classification accuracies between LDA and SVM in all tasks of MI. Table 1 shows the mean of classification accuracies and standard deviation when using LDA and SVM of all tasks. It also indicated that there are no statistically significant differences in accuracy between using LDA and SVM. Thus either LDA or SVM could be chosen as a classifier. Linear classifiers are used to classify between two groups of data. LDA is the most widely used linear classifier [39, 46]. However, SVM is also found to give high accuracies due to its customizable kernel [47]. The comparable performance between LDA and SVM from our experiments conforms to these literature review findings [46–48].

**Fig. 12:**
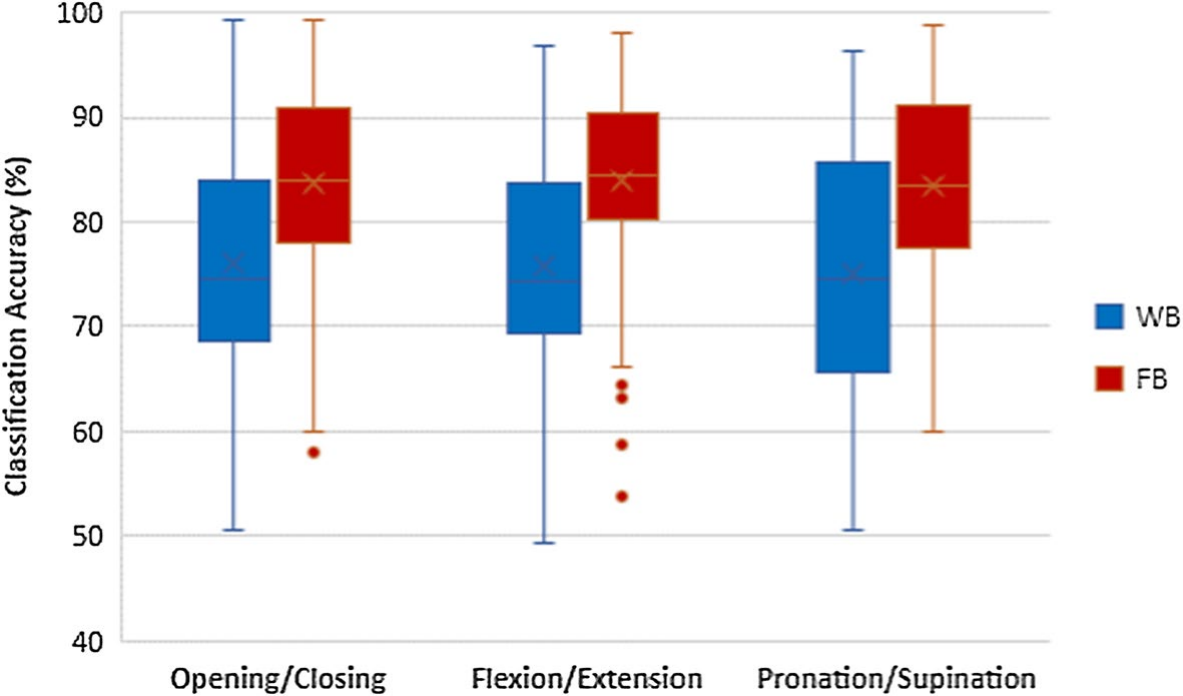
Comparison of WB feature and FB feature

Figure 12 depicts that there are differences in classification accuracies between WB and FB in all tasks of MI. Table 1 also shows the mean of classification accuracies and standard deviation when using WB and FB of all tasks. It also demonstrates that there are statistically significant differences in accuracy using WB and FB.

Considering classification accuracy of using FB and WB feature, due to the increment of features of using FB might increase the likelihood of overfitting, the classification of left hand and right hand of each MI task was performed on a separate validation set. The results showed the classification accuracy on validation set was comparable to the results of eightfold cross validation method. This shows that overfitting does not strongly affect classification accuracy in this case. It is also common to analyze EEG signals in five separate frequency bands as it is believed that each band responds to different brain activities [3]. FB feature extracted these five separate frequency bands and thus gaining more brain activity information. Hence using FB achieved higher classification accuracy then using WB. Furthermore, each person could show effects of sensorimotor functions in different frequency bands [26, 27]. Therefore, higher classification accuracy made FB feature more suitable for classifying left hand and right hand in all MI tasks than WB feature.

Table 2 shows the results of comparing the classification accuracies of the first session and the last session of each task. The results are the differences of classification accuracies between these two sessions. The statistical results which were calculated using ANOVA with Bonferroni correction are also shown in the table.

According to the results of hand opening/closing task as shown in Fig. 8, there were four subjects (S2, S4, S9 and S11) that showed upward trend, but the statistically significant improvements of classification accuracies could be seen in two subjects (S2 and S11).

The result of six subjects (S2, S6, S7, S8, S9 and S10) significantly improved in wrist flexion/extension task. They were six of eight subjects that gave upward trend according to the results illustrated in Fig. 9.

In forearm pronation/supination task, statistically significant improvements of accuracies were found in two subjects (S1). To summarize, from all experiments of MI tasks, nine subjects (S1, S2, S4, S6, S7, S8, S9, S10 and S11) gave significant improvements in accuracy when comparing the first session and the last session. Subject 5 was the only subject that gave downward trend and achieved significantly lower classification accuracy in all MI tasks. Although Subject 3 gave an upward trend in wrist flexion/extension task but the improvement of accuracy was not statistically significant. Subject 3 also gave downward trend with statistically significant decrease of classification accuracy in hand opening/closing task and forearm pronation/supination task. It might be concluded that Subject 3 and Subject 5 did not respond to MI training while other subjects have potential to respond to MI training.

**Fig. 13:**
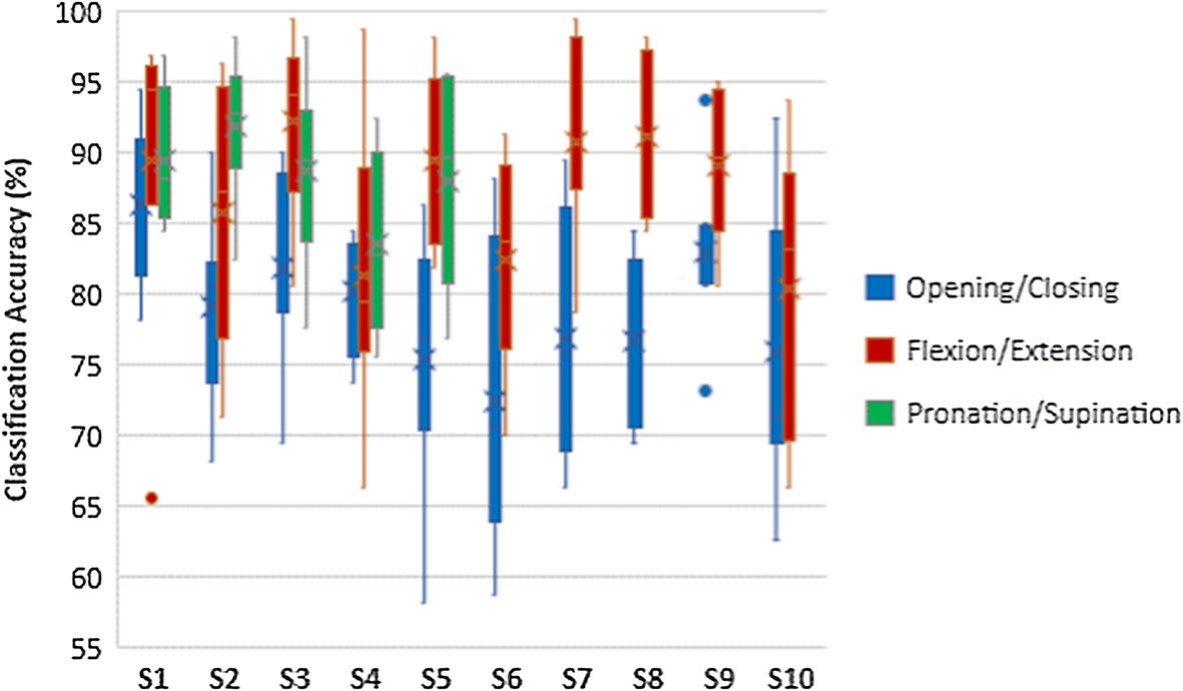
Comparison of classification accuracies of each MI task

Furthermore, the average accuracies of individual sessions of wrist flexion/extension task and forearm pronation/supination task were higher than that of hand opening/closing task as shown in Fig. 13. Familiarity with performing MI tasks could be the cause of these results. It might also be concluded that wrist movements should be considered for MI tasks because the accuracies of wrist movement tasks were higher than that of hand movement task. In addition, the accuracies were consistent with the criterion that was defined in the study by Keng et al. [23]. The capability of performing MI task was assessed by the criterion which stated that the accuracy that participants need to achieve was 60%.

After the experiment was completed, subjects were interviewed. All of them said that the experiment was quite boring and they were sleepy. They were sometimes frustrated when the set up took too much time. Boredom and sleepiness could also contribute to low accuracy results of Subject 5 who admitted drowsiness during sessions. In this study, the minimum set up time was approximately 10 min. The maximum set up time was almost 1 h which took the overall time of that experiment session to almost one and a half hour. Although there is no practical guideline for suitable experimental time, experimental session is approximately 1 h including setup time in most studies [16, 17, 50].

Moreover, lower concentration during experiment might be the cause of downward trend in some subjects. On the other hand, these subjects may simply not be responsive to MI training. Both these results are consistent with previous findings that significant improvement could not be found in some subjects [16, 17]. Thus, to exclude subjects who may not respond to MI training, most of EEG-based studies have screening procedure before their experiments start [16, 17, 23, 35–37].

Since the accuracy also reflects the system classification performance, it could be increased by improvement in classification algorithm [16, 17]. However, since we employed the same classification system throughout our experiments, the accuracy could indicate the change in the subject’s ability to perform MI tasks. We, therefore, use it to analyze trends and compare the effect to training on individual subjects.

The results in Table 3 showed that the accuracies from left hand and right hand were comparable in most subjects. For classifying each MI task of left hand and right hand, the mean of classification accuracies of each task with their standard deviation are shown in Table 4. Paired t-test was used to calculate statistical results. The significant level was set at P-value of 0.05.

**Table 4:** The results of comparing accuracies of left hand and right hand of each MI task

Classification accuracy (%) ± SD					
M1M2		M1M3		M2M3	
Left	Right	Left	Right	Left	Right
84.71 ± 4.93	84.64 ± 5.32	88.38 ± 4.56	87.59 ± 4.97	77.59 ± 3.83*	80.98 ± 2.12*

* P < 0.05

There were not statistically significant differences of accuracies in M1M2 and M1M3. This indicates that being left-handed or right-handed does not affect the person’s capability of performing MI. This could be because all three MI tasks are basic hand, wrist and forearm movements. Hence, subjects should be able do the tasks easily on both left and right limbs. However, the result of classifying left hand and right hand in M2M3 is shown in number with asterisk. This means that statistically significant difference in left and right hand performances was found in M2M3. This finding is in contrast to M1M2 and M1M3, so it would be further investigated.

Moreover, it could be seen that the accuracies of classifying hand opening/closing task and wrist flexion/extension task were comparable to the accuracies of classifying hand opening/closing task and forearm pronation/supination task. This might be because the brain area that corresponds to hand control is not the same area that corresponds to wrist control. According to American electroencephalographic society guidelines [38], the brain area that corresponding to hand control is around C3 and C4 while the area that corresponding to wrist control is closer to the center of the scalp. Consequently, the classification accuracies of wrist flexion/extension and forearm pronation/supination are lower because it was the result of classifying EEG data from the same area of the brain.

Compare to classifying left hand and right hand MI, classifying each MI on a left hand or right hand is a challenge. This is because it processes EEG data from same side of the brain. However, our results showed that it is possible to do the classification.

The classification system developed here would be considered to combine with robotic arm [40, 41] to create an EEG-based stroke rehabilitation system. In this integrated rehabilitation system, the robot arm would support a patient in regaining hand and arm movement. A patient’s EEG would be detected and processed by our system. Previous study found MI and real movement result in the same ERD and ERS [3]. When the patient tries to move his/her limbs in one of the three tasks, our system would detect and send signals to the robot arm. The robot arm would provide an assist or resistance for muscle strength building, depending on the patient’s conditions [40, 41].

## Conclusion

The findings from this work could be used toward system development. From these results, either LDA or SVM can be chosen as a classifier in EEG-based stroke rehabilitation application because their accuracies are not statistically significantly different.

Higher classification accuracy made FB feature suitable for classifying left hand and right hand in all MI tasks than WB feature. The purpose of FB feature in this study is just to study the effect of the increasing number of features to classification accuracy. According to the results, even if feature selection algorithm was not used, the general idea is that the more features used, the better the classification performance. WB feature represents conventional CSP method, whereas FB feature increase the number of features for classifying MI. FB feature gave statistically significantly higher classification accuracy than WB feature. The result shows that even without applying special algorithm, simply increasing features gives higher classification accuracy.

The idea that more training sessions yielded more capabilities of performing MI is supported by the results of trend analysis in nine of eleven subjects. The classification accuracies of all tasks also indicate the possibility of using these three movements as MI tasks in EEG-based stroke rehabilitation application. The accuracies of classifying each MI task of left hand and right hand also indicate the possibility of classifying EEG data from same side of the brain area.

Furthermore, to develop usable EEG-based stroke rehabilitation system, maximum experimental time of 1 h is recommended to avoid boredom, sleepiness and irritability which might lead to lower concentration during the experiment. Number of electrodes is another parameter that should be considered because the parameter will affect overall experimental time. The more electrodes are used, the more time is needed to set up. Our results suggest that eleven electrodes which cover the position of C3 and C4 to the center of the scalp are the number of electrodes that gives good results in MI classification.

For further work, the system will be developed into real-time/online system. Experimental paradigm will be modified. Conventionally, EEG-based stroke rehabilitation system has two experimental sessions. The first session is a calibration session or training session. Online experimental session is the second session. The objective of the calibration session is to create features that will be used to classify MI task in the online session. To get a subject to practice performing MI is another objective of the calibration session. Consequently, adaptive or co-adaptive is the type of system that should be considered because the session variation in EEG data. Adaptive or co-adaptive system constructs EEG features using EEG data from current experimental session together with EEG data from previous calibration sessions. The purpose of this is to relief the effect of the non-stationary characteristic of EEG especially session variation [59–61]. Moreover, in online session, in addition to combining with robotic arm, user interface such as virtual hardware or game play might be needed to give feedback to the subject.

## Abbreviations

BCI: brain computer interface
CSP: common spatial pattern
EEG: electroencephalography
ERD: event-related de-synchronization
ERS: event-related synchronization
FB: filter bank
FBCSP: filter bank common spatial pattern
NBPW: Naïve Bayes Parzen Window
MI: motor imagery
SMRs: sensorimotor rhythms
WB: whole band
